# Differential Incorporation of Carbon Substrates among Microbial Populations Identified by Field-Based, DNA Stable-Isotope Probing in South China Sea

**DOI:** 10.1371/journal.pone.0157178

**Published:** 2016-06-09

**Authors:** Yao Zhang, Wenchao Deng, Xiabing Xie, Nianzhi Jiao

**Affiliations:** State Key Laboratory of Marine Environmental Science & Institute of Marine Microbes and Ecospheres, Xiamen University, Xiamen, 361101, China; CAS, CHINA

## Abstract

To determine the adapted microbial populations to variant dissolved organic carbon (DOC) sources in the marine environment and improve the understanding of the interaction between microorganisms and marine DOC pool, field-based incubation experiments were carried out using supplemental ^13^C-labeled typical substrates *D*-glucose and *D*-glucosamine (*D*-Glc and *D*-GlcN, respectively), which are two important components in marine DOC pool in the South China Sea. ^13^C- and ^12^C-DNA were then fractionated by ultracentrifugation and the microbial community was analyzed by terminal-restriction fragment length polymorphism and 454 pyrosequencing of 16S rRNA gene. ^12^C-DNA-based communities showed relatively high similarities with their corresponding *in situ* communities, and their bacterial diversities were generally higher than ^13^C-DNA-based counterparts. Distinct differences in community composition were found between ^13^C- and ^12^C-DNA-based communities and between two substrate-supplemented ^13^C-DNA-based communities; these differences distinctly varied with depth and site. In most cases, there were more genera with relative abundances of >0.1% in *D*-Glc-incorporating communities than in *D*-GlcN-incorporating communities. The *Roseobacter* clade was one of the prominent actively substrate-incorporating bacterial populations in all ^13^C-DNA-based communities. *Vibrio* was another prominent actively *D*-GlcN-incorporating bacterial population in most incubations. However notably, different OTUs dominated this clade or genus in different treatments at different depths. Altogether, these results suggested that there were taxa-specific differences in DOC assimilations and, moreover, their differences varied among the typical water masses, which could have been caused by the variant compositions of original bacterial communities from different hydrological environments. This implies that ecologically, the levels of labile or recalcitrance of DOC can be maintained only in a specific environmental context with specific bacterial community composition.

## Introduction

Heterotrophic bacteria function as a driving force of ocean carbon cycling, by incorporating, respiring, and transforming various organic substrates supplied into the ocean. Dissolved organic carbon (DOC) composition in the ocean is extraordinarily diverse and, although thousands of organic molecules have been identified, these only account for <10% of marine DOC [1, 2]. Clearly, no single taxon carries out incorporation of such various DOC [3], and there are taxa-specific differences in DOC assimilation [4, 5]. For example, SAR11 can use dimethylsulfoniopropionate but prefers amino acids [6, 7]; aerobic anoxygenic phototrophic bacteria (AAPB) selectively take up DOC generated by phytoplankton [8]; and the Archaea, widely distributed in the deep sea, are proficient in typical deep sea DOC metabolism, such as *D*-amino acid [9]. Such specificity in incorporation of DOC by prokaryotic populations suggests that so-called “labile” substrates are available to some microbes but might be recalcitrant or difficult to metabolize for others [10]. This is ecologically important in the marine environment, implying that the levels of labile or recalcitrance of DOC can be maintained only in a specific environmental context with specific bacterial community composition [11]. Thus, the identity of microbial populations adapted to different DOCs among different water messes will improve the understanding of the interaction between microorganisms and the marine DOC pool.

In the marine DOC pool, neutral sugars and amino sugars are very important components that make up 5%–18% of total DOC in the marine euphotic layer [12] and roughly 2% of total deep-sea DOC [13]. *D*-glucose and *D*-glucosamine (*D*-Glc and *D*-GlcN, respectively) are important components of neutral sugars and amino sugars, respectively, as well as also being important materials for bacterial cell structures. *D*-Glc is prevalent in the marine environment and supports 15−47% of bacterial production [14, 15]. *D*-GlcN is a structural element of the polysaccharides chitosan and chitin, which compose exoskeletons of crustaceans and other arthropods, as well as cell walls of fungi and many higher organisms. It has also been found to be an important carbon and nitrogen source for marine bacteria [16–19]. As *D*-GlcN only differs from *D*-Glc by the former having an amine group, their structures are quite similar. However, their bioavailability could be substantially different. So far, differences in microbial populations incorporating these two typical substrates among different water masses, such as coastal versus sea-basin waters and shallow versus deep waters, remain unclear. Investigation on the microbial populations adapted to these two typical substrates in the marine environment may be a breakthrough for attempting to understand the interaction between microorganisms and the marine DOC pool.

The bacterial populations which assimilate a specific DOC substrate have been exactly identified by tracking substrate incorporation into biomass [20]. Many studies have been performed using radiolabeled compound assimilation and phylum-level phylogenetic probes to assign uptake of compounds to a microbial group [3, 9, 21, 22]. However, the results from such phylum-level diversity assessments have been too coarse to discern subtle spatial or temporal phenotypic differences [23]. Stable isotope probing (SIP), first proposed by Radajewski et al. [24], is a cultivation-independent technique that tracks incorporation of substrates labeled with a stable isotope into cell biomass. As DNA contains the most taxonomical information, DNA-based SIP (DNA-SIP) combined with high-throughput sequencing offers the ability to identify a broad spectrum of microorganisms involved in a particular process and with high phylogenetic resolution [20, 25]. In the present study, field-based incubation experiments with the supplemental ^13^C-labeled *D*-Glc and *D*-GlcN were carried out in the South China Sea (SCS) and, subsequently, ^13^C- and ^12^C-DNA were analyzed by terminal-restriction fragment length polymorphism (T-RFLP) and pyrosequencing. The objectives of this study were to identify microbial populations that incorporate these two ^13^C-labeled substrates; compare the results among typical water masses of the SCS; and evaluate taxa-specific or environment-specific bioavailability of the substrates. These results will provide great insight for a better understanding of the interaction between microorganisms and marine DOC pool.

## Materials and Methods

### Study Station and Water Collection

The SCS is one of the largest marginal seas, with a deep basin in the tropical-subtropical western North Pacific [26]. The Ministry of Foreign Affairs, State Oceanic Administration, and Ministry of Transport of the People’s Republic of China issued the permission for field studies in the SCS. The South East Asia Time-series Study station (SEATS, 116°N, 18°E) of the SCS central basin, with water depth of 3850 m, is characterized by oligotrophic water [27]. D001 station (110.72°N, 18.97°E), located near Hainan Island, is characterized by relatively eutrophic water and was undergoing an upwelling event during sampling. Field-based incubation experiments with ^13^C-labeled *D*-Glc and *D*-GlcN supplements were conducted at the above two stations during a summer research cruise from July 28^th^ to August 25^th^, 2012. Seawater was collected in Niskin bottles from 5, 200, 800, and 3000 m depth at the SEATS station and 0, 25, and 70 m depth (near the bottom) at the D001 station by CTD.

### Field-Based Incubation Experiments

Forty liters of seawater collected from each layer was immediately filtered through a 3-μm pore-size, 293-mm diameter polycarbonate filter for removals of grazers and macrophytoplankton. Filtered seawater was used to leach 20 L polycarbonate bottles for three times before incubation experiments, which were previously washed with 10% HCl solution and Millipore-Q water. All incubations had two treatments and no replications. A 17-L volume of filtered seawater at SEATS and 4-L at D001 was supplemented with 99% ^13^C-labeled *D*-Glc or *D*-GlcN to produce a final ^13^C concentration of 100 μM in the polycarbonate bottles. All bottled samples were covered by aluminum foil and incubated at a steady temperature of ~27°C in the shipboard laboratory. After 3 d, all of the incubated seawater was filtered through 0.2-μm pore-size, 47-mm diameter polyethersulfone filters (Millipore Sterivex filters, EMD Millipore Corp., Merck KGaA, Darmstadt, Germany) with a suction pressure of <0.03 MPa. Two liters of seawater were filtered to collect the *in situ* communities at the same sites. All filters were then flash-frozen in liquid nitrogen for 10 min and subsequently stored at −80°C until DNA extraction in laboratory.

### DNA Extraction and Quantification

A total of ten incubation samples and five *in situ* samples (2–4 L/sample) were subject to DNA extraction according to the phenol-chloroform-isoamyl alcohol method [28]. Purified DNA was checked with a NanoDrop device (ND2000, Thermo Fisher Scientific, Inc., Waltham, MA, USA), and the DNA concentration fluorometrically quantified [29] with QuantiFluor^™^ dsDNA system (Promega Corp., Madison, WI, USA). DNA of *in situ* samples were obtained only for 5 m of site SEATS and 0 and 25 m of site D001, while DNA extraction failed for other two samples due to being stored in RNAlater. ^12^C/^13^C-DNA of *Escherichia coli* was obtained as described by Dumont et al. [30] as a positive standard after incubation with ^13^C-labeled *D*-Glc as the sole-carbon-source.

### CsCl Ultracentrifugation and Gradient Fractionation

Approximately 3 μg of DNA from each sample was mixed with a gradient buffer, containing 0.1 M Tris, 0.1 M KCl and 1 mM EDTA, and then the mixture added to a CsCl solution (1.89 g∙mL^-1^), to form a final density of 1.723–1.725 g∙mL^-1^ for ultracentrifugation and gradient fractionation following the protocol of Neufeld et al. [31]. Ultracentrifugation conditions were 140,000×g (~37,700 rpm) in a vertical rotor (VTi 65.2, Beckman Coulter, Inc., Brea, CA, USA) at 20°C for 69 h under vacuum [32]. After centrifugation, mineral oil was injected into the top of each 5.1 mL ultracentrifuge tube by a syringe pump (Braintree Scientific INC., Braintree, MA, USA) with a uniform flow of 425 μL∙min^-1^. During the oil injection, the DNA/CsCl mixture was collected from the tube bottom in 12 sterile 1.5 mL tubes, resulting in 12 density gradient fractions; the densities of all fractions were determined with a refractometer. DNA in each fraction was then precipitated by adding two volumes of PEG solution (30% PEG 6000, w/v, 1.6 M NaCl, and 20–40 μg of glycogen) and then resuspended in 35 μL of TE (10 mM Tris-HC1, 1 mM EDTA, pH 8.0) and fluorometrically quantified as described above.

### T-RFLP Fingerprinting Analysis

The 2^nd^–11^th^ fractions of each sample (10 samples; total 100 fractions) were analyzed with T-RFLP fingerprinting as described by Zhang et al. [33]. Briefly, the fluorescently labeled forward primer 27F (5' [6FAM]-AGAGTTTGATCMTGGCTCAG-3') and the unlabeled reverse primer 927R (5'-ACCGCTTGTGCGGGCCC-3') were used to amplify bacterial 16S rRNA genes [34, 35]. PCR products were checked by electrophoresis on 1% agarose gel and purified with an agarose gel DNA purification kit (Tiangen Biotech Co., Ltd., Beijing, China). Purified products were digested with restriction enzyme FastDigest RsaI (Thermo Fisher Scientific, Inc.) at 37°C for 1–2 h. The digested products were recovered using 20 uL of sterile deionized water and ethanol precipitation. Purified products were then mixed with 0.5 uL of an internal size standard (ET ROX-900) and then detected using a MegaBACE genetic analyzer (Amersham Biosciences Corp., Piscataway, NJ, USA). The output was transferred to T-REX software (http://trex.biohpc.org/) [33, 36] for noise removal and construction of a data matrix. The obtained matrix was further analyzed with Primer 5 analysis software to determine fragment profiles of the 12 density gradient fractions from each experimental sample as well as community similarity between fractions. Typical ^13^C- and ^12^C-fractions were chosen for pyrosequencing.

### PCR Amplifying of V1–V3 Region of Bacterial 16S rRNA Gene and 454 Pyrosequencing

PCR reaction mixtures and amplifying conditions for the V1–V3 region of bacterial 16S rRNA genes were in accordance with Zhang et al.[37], using the universal primers 27F (5'-AGAGTTTGATCCTGGCTCAG-3') and 534R (5'-ATTACCGCGGCTGCTGG-3'). In addition, a unique 10 bp barcode was used to tag each sample to enable multiplex sequencing. Each sample was amplified in triplicate to weaken the influence of specific amplification and with a negative control for each barcoded primer pair to verify samples were uncontaminated. Triplicate PCR products from each sample were combined, checked by electrophoresis on 1% agarose gel and purified with an agarose gel DNA purification kit (Takara Bio Inc., Otsu, Japan). The quantity and quality of the combined products were checked using a NanoDrop device (ND-2000, Thermo Fisher Scientific, Inc.). Pyrosequencing was carried out on a 454 Genome Sequencer GS-FLX Titanium instrument (Roche-454, Life Sciences, Branford, CT, USA) at the Chinese National Human Genome Center (Shanghai, China).

### Sequence Analysis

The criteria previously described [38] were used to assess the quality of sequence reads. We eliminated sequences that contained more than one ambiguous nucleotide (N), that did not have a complete barcode and primer at one end, or that were shorter than 200 bp after removal of the barcode and primer sequences. The remaining sequences passing the pipeline filters were assigned to samples by examining the barcode. Libraries of sequences and operational taxonomic units (OTUs) were analyzed in MOTHUR following the standard operating procedure (www.mothur.org/wiki/Schloss_SOP) [39]. Briefly, the sequences were simplified to unique sequences with “unique.seqs” command. The obtained unique sequences were aligned to the SILVA bacterial database [40] using “align.seqs” command. Then, “screen.seqs” command was applied to remove those sequences whose lengths were outside the desired range and “filter.seqs” command applied to remove those columns with only gaps. Those sequences within 2 bp of difference to a more abundant sequence were merged with “pre.cluster” command for reducing sequencing error. Chimeras were identified and removed using “chimera.uchime” and “remove.seqs” commands, respectively. These processes avoided superfluous computations, as much as possible, for the following commands below. Finally, classification was carried out using the MOTHUR version of the “Bayesian” classifier with the SILVA reference sequences and taxonomic outline. The confidence cut-off was set to 60%. The sequences that were classified as “Cyanobacteria_Chloroplast” or “Mitochondria” or could not be classified at the kingdom level were removed from the data set.

Sequences were further clustered into Operational Taxonomic Units (OTUs) using the furthest-neighbor algorithm and the cutoff value set at 0.03 [41]. All the samples were rarefied to an equal number of sequences using “sub.sample” command for normalization. Based on OTU assignments, library richness and diversity indices (ACE, Coverage, Chao, and Shannon) were calculated using “summary.single” command, and rarefaction curves were calculated at 0.03 distance cutoff using “rarefaction.single” command. To determine the community similarity between fractions, dendrograms were generated based on Bray-Curtis similarities of OTU relative-abundance matrices using group average model of Primer 5 cluster function [42].

### Phylogenetic Tree Construction

Representative sequences from each OTU that fell in the most prominent actively substrate-incorporating bacterial populations (*Roseobacter* and *Vibrio* in the present study) and its relative abundance was >1% of total reads in any ^13^C-DNA library were phylogenetically analyzed. Sequences were compared to known 16S rDNA sequences in the database using the BLASTN search (http://www.ncbi.nlm.nih.gov/BLAST/) to aid the selection of the closest reference sequences. Sequences were aligned and compiled using the MEGA5 program and neighbor-joining phylogenetic trees were constructed.

### Genbank Accession Numbers

454 sequencing data was deposited in the NCBI Sequence Read Archive under study accession number SRP066082. The representative sequences used in the phylogenetic trees are deposited in GenBank under accession numbers KU836758 to KU836853.

## Results

### Distribution of DNA in CsCl Density Gradients

**Fig 1:**
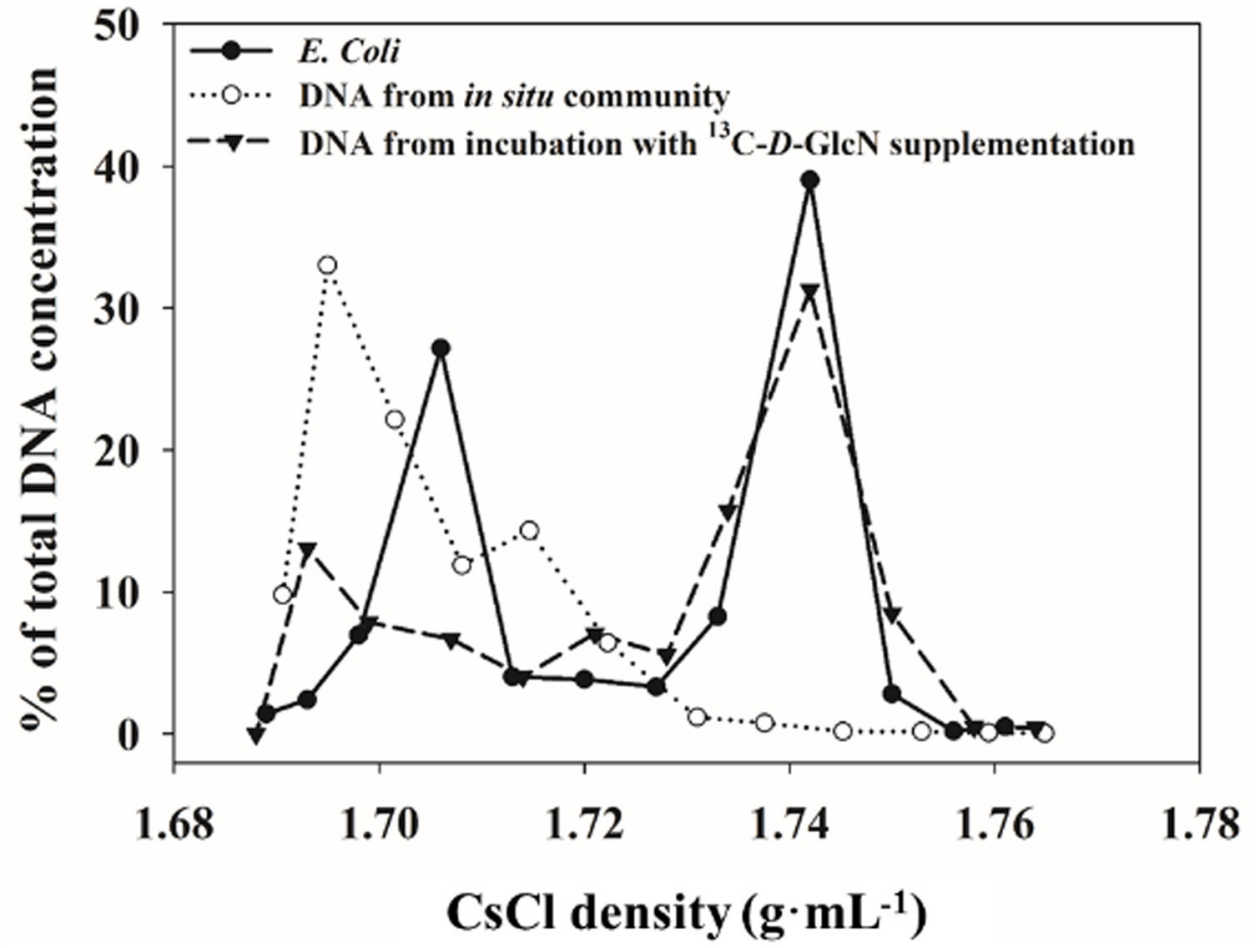
Distribution of DNA in a CsCl density gradient, produced from surface water of site D001 after incubation with supplemental ^13^C-labeled *D*-GlcN. Distribution of corresponding natural DNA shown as a negative control. Mixture of ^12^C- and ^13^C-DNA from *Escherichia coli* shown as a positive standard.

For locating ^13^C- and ^12^C-DNA in CsCl density gradients, mixtures of ^12^C- and ^13^C-DNA extracted from positive control *E*. *coli* was fractionated by ultracentrifugation and the DNA distribution in a CsCl density gradient is shown in Fig 1. There were two peak DNA concentrations at densities of 1.706 and 1.742 g∙mL^-1^, which corresponded to the theoretical density values of ^12^C and ^13^C-DNA of *E*. *Coli*, respectively, calculated using the following formula:

ρ = (0.098 [G+C]) + 1.66

[43].

**Fig 2:**
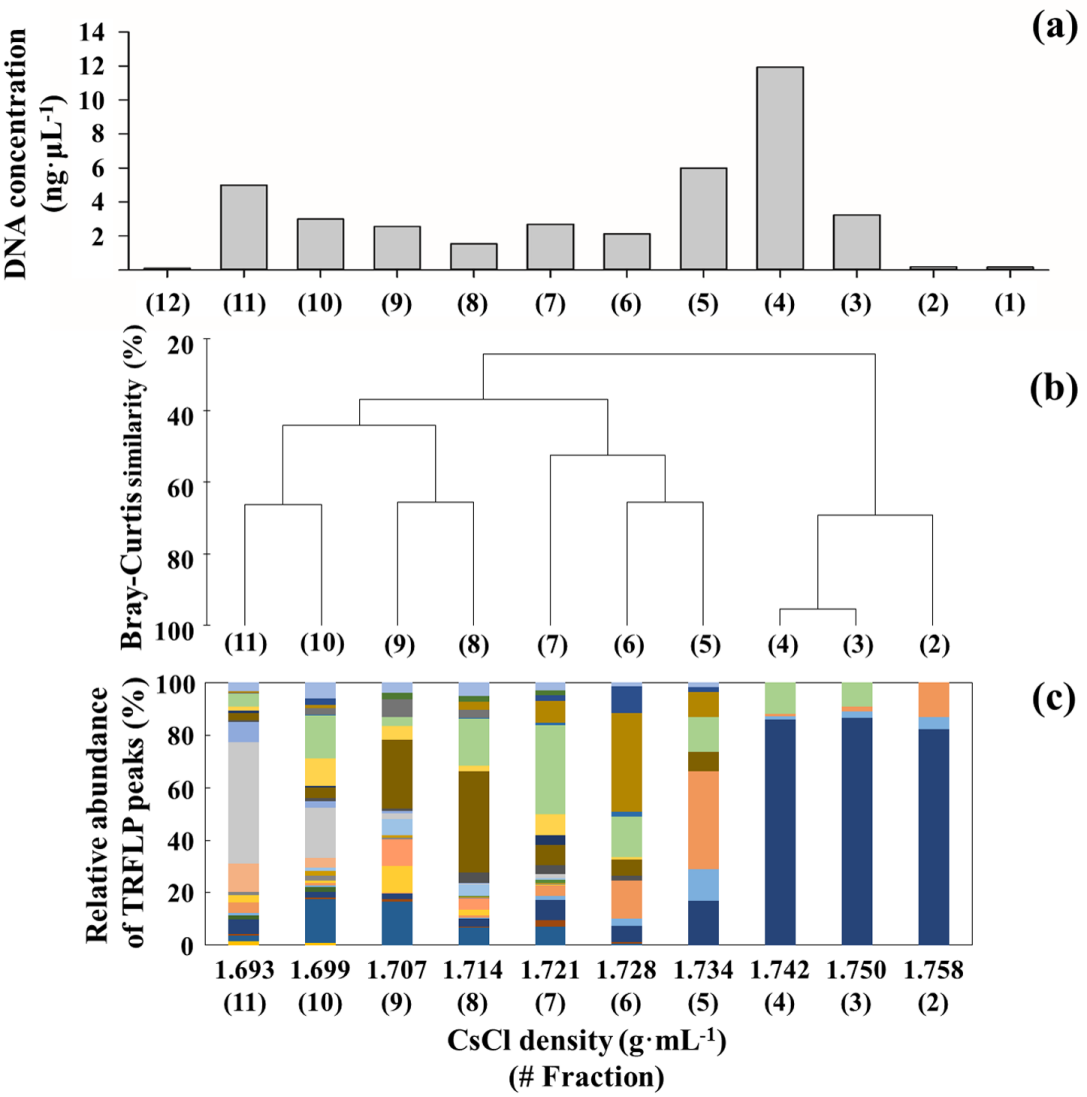
The 2^nd^ (heavy) to 11^th^ (light) density gradient fractions were analyzed with T-RFLP among 12 density gradient fractions produced from ultracentrifugation of DNA from surface water of site D001 after incubation with supplemental ^13^C-labeled *D*-GlcN. (a) Distribution of DNA concentrations in CsCl density gradients; (b) Relative abundance of T-RFLP peaks (color bars) in each DNA fraction; (c) Community similarity analysis based on T-RFLP fingerprinting.

The all ^13^C-DNA from incubations with ^13^C-labeled substrate supplementation peaked in the density range from 1.73 to 1.743 g∙mL^-1^, corresponding to the 4^th^–5^th^ density fractions, while ^12^C-DNA peaked from 1.693 to 1.705 g∙mL^-1^, corresponding to the 10^th^–11^th^ fractions (Figs 1 and 2a, S1 Table).

### Bacterial Community Composition Based on T-RFLP Fingerprinting

Among the 12 density gradient fractions, the 2^nd^ (heavy) to 11^th^ (light) density gradient fractions were analyzed with T-RFLP. In general, the community structure showed distinct differences between heavy and light fractions of each sample. In the ^13^C-*D*-GlcN supplemented seawater-incubation experiment at 0 m of site D001, for instance, the cluster analysis of communities from the 10 density gradient fractions revealed three distinct groups at 44.07% Bray-Curtis similarity level: heavy group (2^nd^–4^th^ fractions, Bray-Curtis similarity = 69.33%), light group (8^th^–11^th^ fractions, 44.07%), and middle group (5^th^–7^th^ fractions, 52.52%; Fig 2b and 2c). Based on community similarity analyses and DNA concentration distributions in CsCl density gradients (Fig 2a) [20], two DNA fractions with peak values in the heavy (Heavy 2, H2; e.g., the 4^th^ fraction in Fig 2a) and light (Light, L; the 10^th^ fraction) density ranges, the typical middle DNA (Middle, M; the 7^th^ fraction) between them, and the extremely heavy DNA (Heavy 1, H1; the 2^nd^ fraction) were selected for 454 pyrosequencing and further analysis. The selected density fractions for each sample according to the same means are shown in S1 Table. As sequences analyses indicated that H2s were the most representative of populations incorporating ^13^C-labeled substrates, only the H2 fraction from each sample was selected and analyzed together with the light fraction.

### Comparison between ^13^C- and ^12^C-DNA-Based Bacterial Communities

The 454 sequencing coverage of samples from sites SEATS and D001 ranged from 79.5% to 97.2% (average, 90.1%) and from 74.4% to 94.5% (average, 88.6%), respectively. Diversity indexes (ACE, Chao, Shannon, and Simpson) showed that the bacterial diversity of light fractions (L), except for the incubation with supplemental ^13^C-labeled *D*-Glc at 0 m of site D001, were generally higher than heavy fractions (H2s) in each incubation (S2 and S3 Tables). The results of rarefaction curves also showed a similar phenomenon (data not shown).

**Fig 3:**
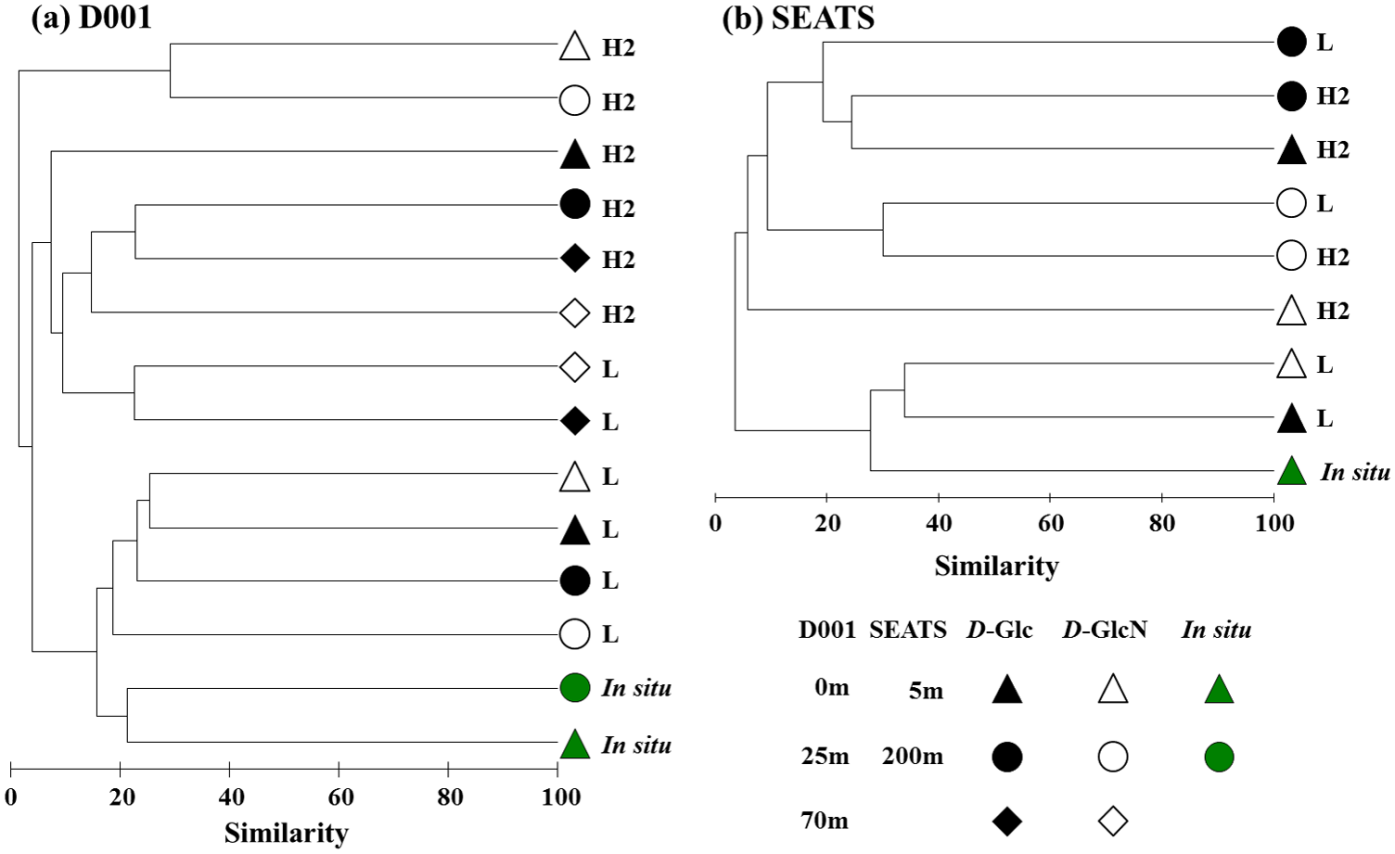
Dendrogram constructed using group average model based on Bray-Curtis similarities between communities at D001 (a) and SEATS (b) sites.

Heavy fractions representing populations actively incorporating ^13^C-labeled substrates and light fractions representing populations not incorporating ^13^C-labeled substrates were analyzed with dendrogram. At the coastal site D001, ^12^C-DNA-based communities clustered together with *in situ* communities, there being two subclusters at 0–25 m and 70 m, respectively. In general, ^13^C-DNA-based communities clustered separately according to the two supplemented ^13^C-labeled substrates, but *D*-Glc-incorporating communities at 0 m separated from those at 25 and 70 m and *D*-GlcN-incorporating communities at 70 m separated from those at 0 and 25 m (Fig 3a). At sea-basin site SEATS, the cluster pattern was also co-indicated by both the supplemental ^13^C-labeled substrates and water depth (Fig 3b). In general, the communities clustered separately according to supplemental ^13^C-labeled substrates, except for the surface water (5 m) where two ^12^C-DNA-based communities and the *in situ* community clustered together. In addition, ^12^C-DNA-based community at 200 m separated from ^13^C-DNA-based communities at 5 and 200 m within the *D*-Glc cluster; *D*-GlcN-incorporating communities at 5 m distinctly different from that at 200 m within the *D*-GlcN cluster (Fig 3b).

### Actively Substrate-Incorporating Bacterial Populations

**Fig 4:**
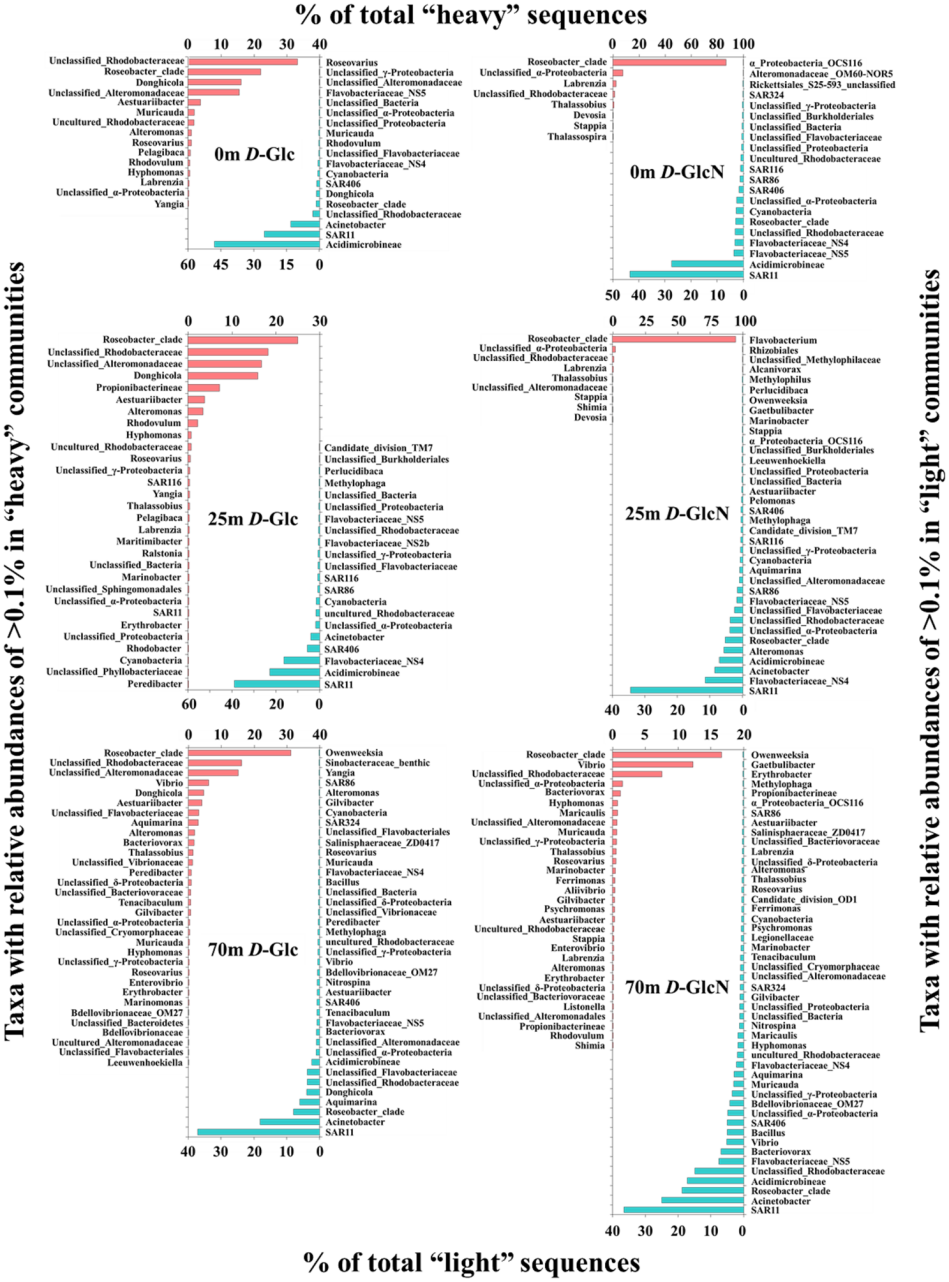
Taxa with relative abundances of >0.1% of total sequences in ^13^C-DNA-based (left axis) and ^12^C-DNA-based (right axis) communities at site D001.

Further analyses focused on actively substrate-incorporating bacterial taxa. The *Roseobacter* clade and many unclassified taxa belonging to *Rhodobacteraceae* and *Alteromonadaceae* (>10% of total ^13^C-DNA sequences) were the most abundant populations incorporating *D*-Glc at all three depths for site D001. *Rhodobacteraceae Donghicola* also accounted for >10% of ^13^C-DNA sequences at 0 and 25 m. For *D*-GlcN-incorporating organisms, the *Roseobacter* clade was a highly active population, accounting for >80% of total ^13^C-DNA sequences at 0 and 25 m and >15% at 70 m of site D001. *Vibrio* also accounted for >10% at 70 m. It was clear that there were more genera with relative abundances of >0.1% in *D*-Glc-incorporating communities than in the *D*-GlcN-incorporating communities (Fig 4). However, there were more genera with relative abundances of >0.1% in ^12^C- than ^13^C-DNA-based communities, except for the ^13^C-*D*-Glc incorporating community at 25 m. SAR11 was almost the most dominant group in these ^12^C-DNA-based communities.

**Fig 5:**
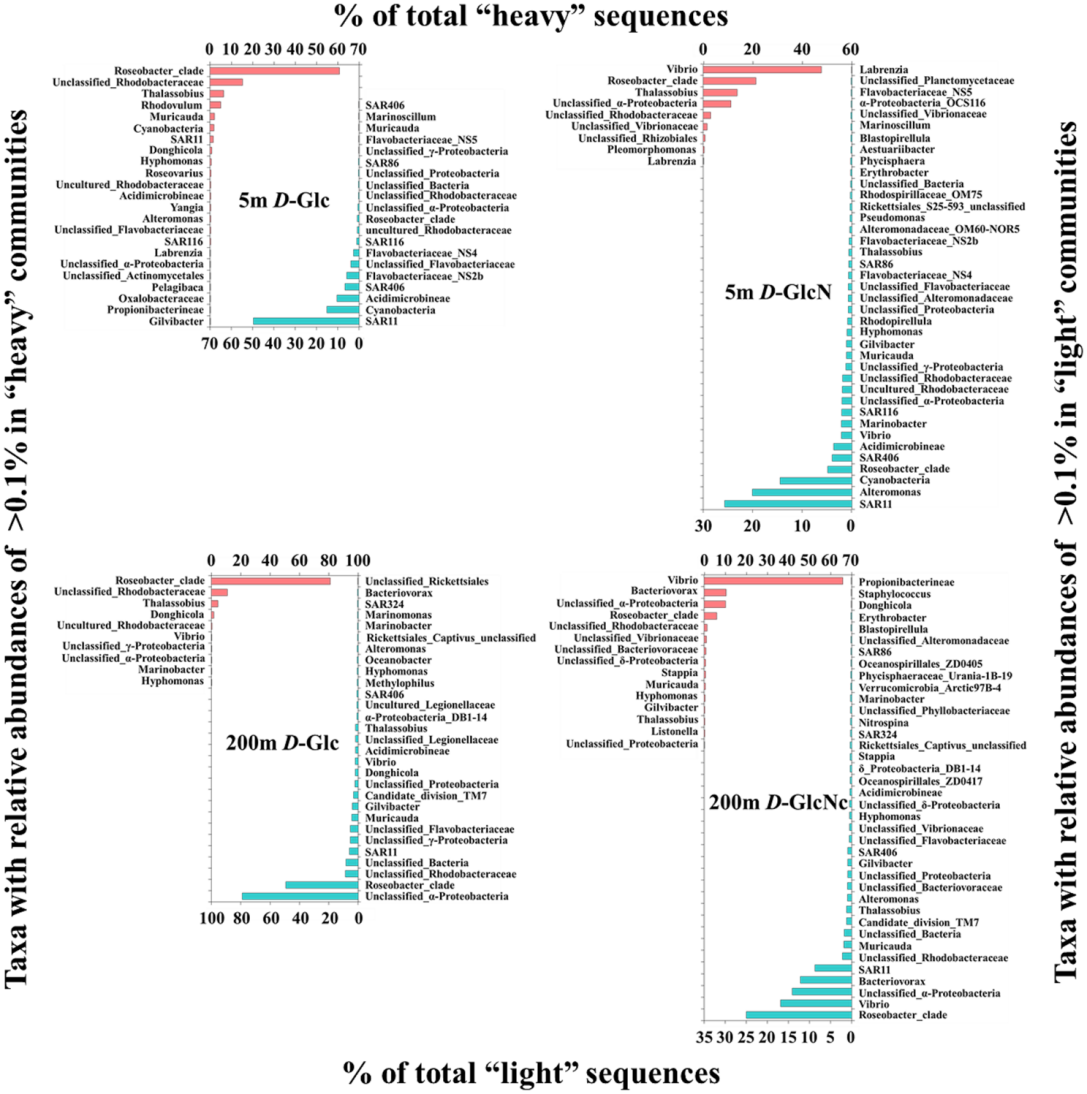
Taxa with relative abundances of >0.1% of total sequences in ^13^C-DNA-based (left axis) and ^12^C-DNA-based (right axis) communities at site SEATS.

At sea-basin site SEATS, the *Roseobacter* clade (>60% of total ^13^C-DNA sequences) and many unclassified taxa belonging to *Rhodobacteraceae* (>10%) were the most abundant populations incorporating *D*-Glc at 5 and 200 m depths. However, for *D*-GlcN-incorporating organisms, *Vibrio* was the most abundant population (>40% of total ^13^C-DNA sequences) at the two depths. The *Roseobacter* clade and *Thalassobius* (>10%) were also relatively active at 5 m. There were more genera with relative abundances of >0.1% in *D*-Glc-incorporating communities than in *D*-GlcN-incorporating communities at 5 m, and on the contrary at 200 m (Fig 5). There were more genera with relative abundances of >0.1% in ^12^C- than ^13^C-DNA-based communities, except for ^13^C-*D*-Glc incorporating community at 5 m. SAR11 and *Cyanobacteria* were dominant groups in ^12^C-DNA-based communities at 5 m, while the *Roseobacter* clade, *Vibrio*, and many unclassified taxa were relatively dominant at 200 m.

### Phylogenetic Analysis of Dominant OTUs within Actively Substrate-Incorporating Bacterial Populations

**Fig 6:**
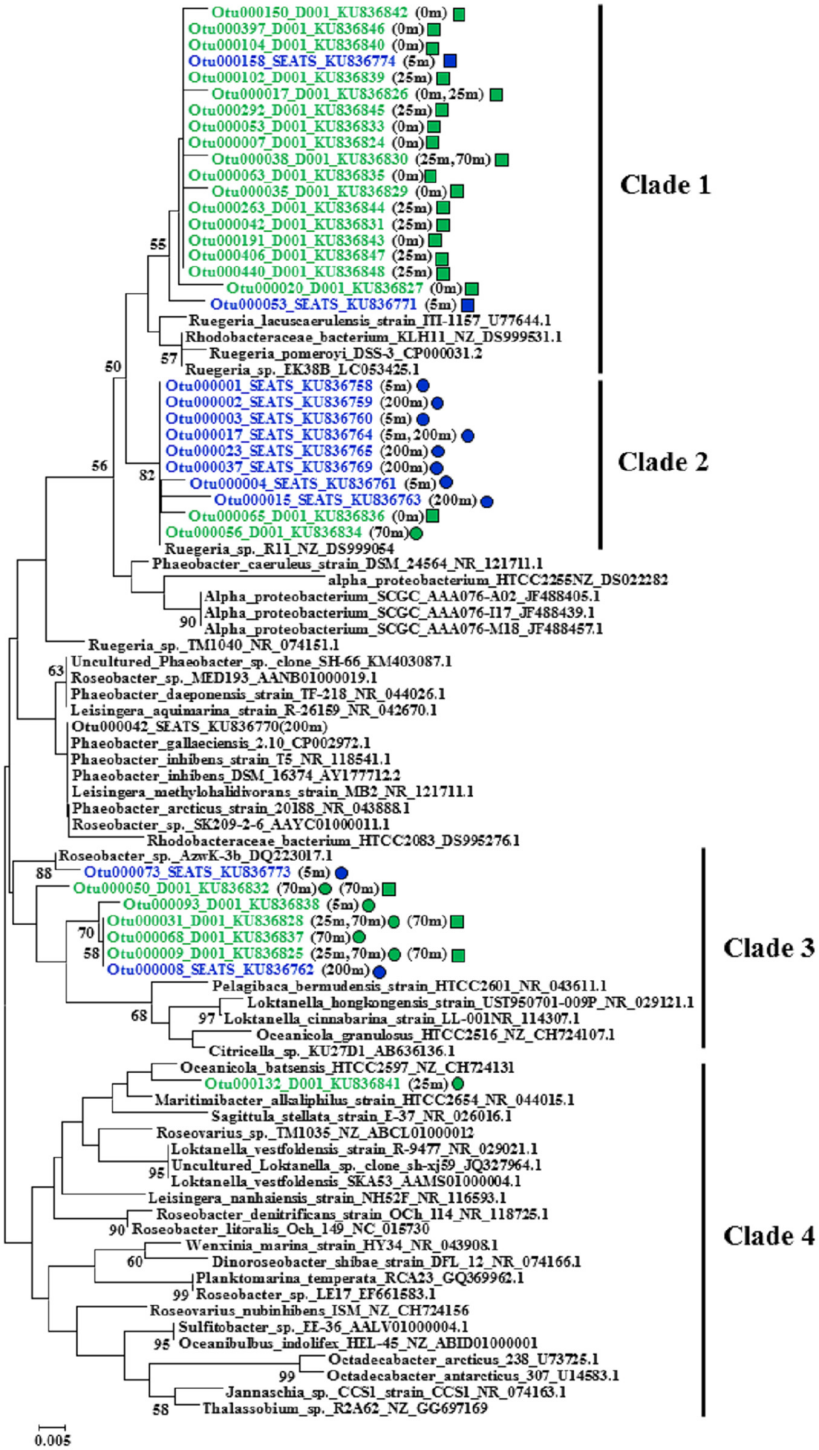
Phylogenetic tree of representative sequences from dominant OTUs that fell in the *Roseobacter* clade constructed using neighbor-joining method. Sequences from this study are shown in blue (site SETAS) and green (site D001). Sampling depths are indicated in brackets. Square indicates ^13^C-*D*-GlcN supplemented incubation. Circle indicates ^13^C-*D*-Glc supplemented incubation. The topology of the phylogenetic tree was evaluated by bootstrap re-sampling method with 1,000 replicates, and bootstrap values greater than 50% are shown.

**Fig 7:**
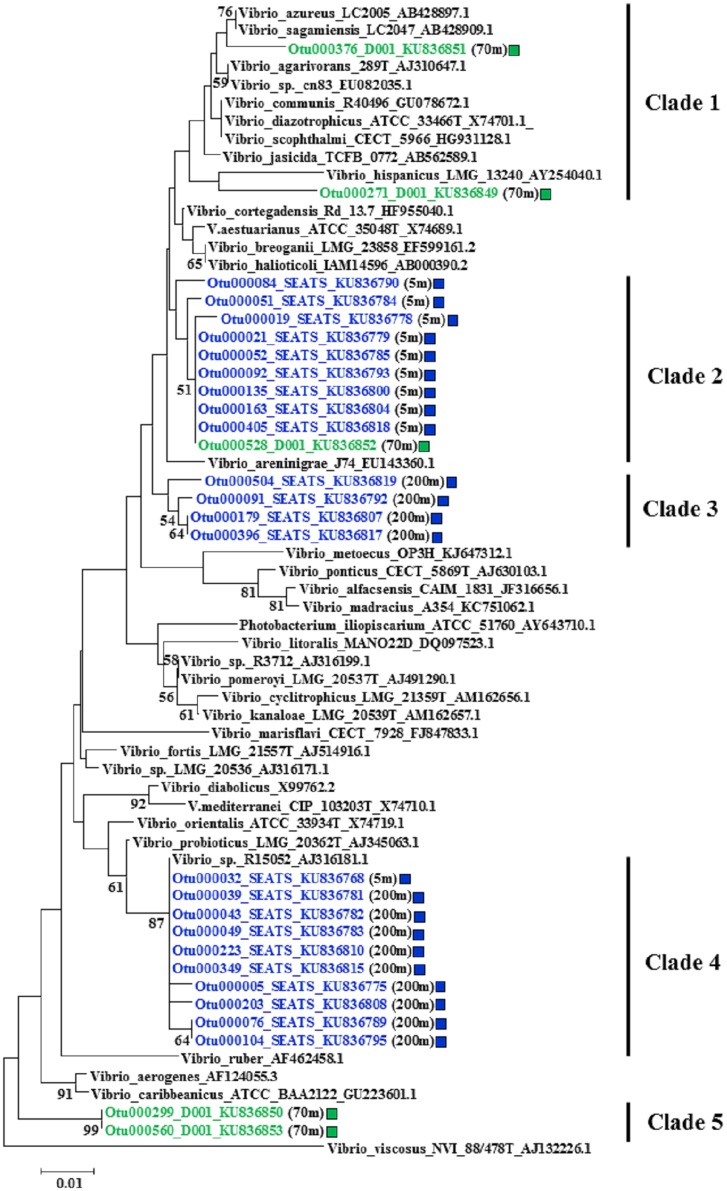
Phylogenetic tree of representative sequences from dominant OTUs that fell in the genus *Vibrio* constructed using neighbor-joining method. Sequences from this study are shown in blue (site SETAS) and green (site D001). Sampling depths are indicated in brackets. Square indicates ^13^C-*D*-GlcN supplemented incubation. The topology of the phylogenetic tree was evaluated by bootstrap re-sampling method with 1,000 replicates, and bootstrap values greater than 50% are shown.

The *Roseobacter* clade was one of the prominent actively substrate-incorporating bacterial populations in all ^13^C-DNA-based communities. *Vibrio* was another prominent actively *D*-GlcN-incorporating bacterial population at site SEATS and 70 m of site D001. Within these two populations, representative sequences from OTUs with relative abundance of >1% of total reads in any ^13^C-DNA library was further phylogenetically analyzed. Interestingly, different OTUs dominated the *Roseobacter* clade or the *Vibrio* genus in different treatments at different depths (Figs 6 and 7). Almost all *Roseobacter* sequences from the ^13^C-*D*-GlcN supplemented incubation at 0 to 25 m and 70 m of site D001 fell in Clade 1 and 3, respectively. Sequences from the ^13^C-*D*-Glc supplemented incubation at the three depths of site D001 distributed in Clade 2, 3, and 4. Sequences from the ^13^C-*D*-Glc supplemented incubation at 5 and 200 m of site SEATS fell in Clade 2 and 3. Sequences from the ^13^C-*D*-GlcN supplemented incubation at 5 m of site SEATS fell in Clade 1 (Fig 6). Similarly, almost all *Vibrio* sequences from the ^13^C-*D*-GlcN supplemented incubation at 5 m of site SEATS fell in Clade 2, and sequences from 200 m fell in Clade 3 and 4. Sequences from the ^13^C-*D*-GlcN supplemented incubation at 70 m of site D001 sporadically distributed in Clade 1, 2, and 5 (Fig 7).

## Discussion

As sufficient ^13^C-labeled DNA was necessary for follow-up laboratory procedures, three conditions were controlled within a reasonable range to obtain enough bacterial cells that had incorporated the offered isotopically-labelled substrate during incubation. First, relatively high concentrations of ^13^C-labeled substrates (100 μM ^13^C) were used in incubation experiments for stimulating cellular growth. However, high substrate concentrations might lead to a fundamentally different metabolic characteristic of DOC incorporation compared with *in situ* or lower concentrations [12, 44]. Even so, DNA-SIP represents an essential first step towards characterizing the active taxa in marine water [44]. Furthermore, ^12^C-DNA-based communities showed relatively high similarities with their corresponding *in situ* communities after incubation suggesting that the results on differential incorporation of ^12^C vs. ^13^C into microbial community are reliable. In addition, the isotope fractionation effect in the incubation systems are neglectable in the concentrations of supplemented isotopic substrates. Secondly, the incubation time was set at 3 d, during which a cross-feeding effect might have occurred among the organisms. However, 3 d of incubation time has been normally used in seawater SIP experiments for obtaining sufficient ^13^C-labeled DNA [20, 44]. In addition, the DNA distribution in a CsCl density gradient after ultracentrifugation could be disturbed by variant genomic G+C content and unlabeled DNA could also be smeared across the density gradient [20, 31]. However, these perturbations would not generally affect the locations of DNA peaks in a CsCl density gradient.

Higher bacterial diversity in ^12^C- than ^13^C-DNA-based communities in each incubation suggested that microbial populations that were adapted to the supplemented organic substrate accounted for only a small part of total bacterial community composition. Also, ^12^C-DNA-based communities showed relatively high similarities with their corresponding *in situ* communities and were distinctly separated from ^13^C-DNA-based communities. As revealed by the dendrograms, there were distinct differences in community composition between ^13^C-DNA-based communities in incubations supplemented with ^13^C-labeled *D*-Glc and *D*-GlcN (Fig 3). This suggested that different ^13^C-labeled substrates were incorporated by different bacterial communities containing variant taxa, such that there were distinctly taxa-specific differences in DOC assimilation. Moreover, their differences varied among the typical water masses, which could have been caused by different original bacterial communities and hydrological environments. Thus, DOC bioavailability was taxa-specific or environment-specific in an ecological environment. Environmental factors, such as temperature, salinity, nutrients, and DOC pool, could influence bacterial-selective substrate incorporation [16, 20].

In most cases, there were more genera with relative abundances of >0.1% in ^12^C-DNA-based communities, in which SAR11 was dominant, than in ^13^C-DNA-based communities, within which more genera (relative abundances of >0.1%) were found in *D*-Glc-incorporating communities than in *D*-GlcN-incorporating communities. The *Roseobacter* clade was one of the prominent actively substrate-incorporating bacterial populations in ^13^C-DNA-based communities. This result was consistent with the conclusion that *Roseobacter* dominates the incorporation of high concentration glucose and amino acids in the German Bay of the North Sea [3, 45]. Thus far, there has been no evidence showing that *Roseobacter* must bloom when incubated because of a “bottle effect” [20]. Genomic analysis of *Roseobacter* has revealed that they have versatile mechanisms for energy and carbon acquisition in various environments, behaving as an “ecological generalist” [46–48]. This might have been one of the reasons that *Roseobacter* thrived in all of the present incubations. It was clear that *Roseobacter* might have played an important role here in DOC transformation and the oceanic carbon cycle. However notably, different OTUs dominated this clade in different incubations from different depths.

*Vibrio* was another prominent actively *D*-GlcN-incorporating bacterial population, as expected. *Vibrio* is an important oceanic chitinolytic bacteria, depolymerizing chitin with cell surface hydrolases to GlcNAc for better use [17]. Previous research has shown that bacterial GlcNAc uptake is suppressed to some extent by glucosamine in seawater [17], suggesting that bacteria might use the same system to transform these two structurally similar amino sugars. These sugars are abundant components of marine organic matter [17], acting as carbon, energy, and nitrogen sources. Therefore *Vibrio* plays a significant role in oceanic nutrient cycling [49]. However, *Vibrio* did not thrived at 0 and 25 m of site D001 among all ^13^C-*D*-GlcN supplemented incubations of the two sites. This could be caused by distinctly different original bacterial communities (Fig 3), which was composed of relatively more actively *D*-GlcN-incorporating bacterial populations as revealed by Fig 4. In addition, similar to *Roseobacter*, different OTUs dominated this genus in different treatments at different depths. These indicated although *Roseobacter* and *Vibrio* thrived in most incubations, there were still distinctly taxa-specific differences in DOC assimilation among different environments.

Altogether, there were significant differences in actively substrate-incorporating bacterial populations between incubations supplemented with ^13^C-labeled *D*-Glc and *D*-GlcN and among different water masses. Although there are only tiny structural differences between glucose and glucosamine, their bioavailability was substantially different and found to be taxa-specific and environment-specific in ecological environments. This is ecologically important, implying that the levels of labile or recalcitrance of DOC can be maintained only in a specific environmental context with specific bacterial community composition. Further study is expected to implement incubation experiments with low supplemental substrate concentrations in a large *in situ* system, such as a mesocosm, for improved detection of microbial selective use of carbon sources in a more or less completely natural environment.

## Supporting Information

S1 Table Density fractions selected for 454-pyrosequencing analysis among the 12 density gradient fractions of each sample. (DOC)

S2 Table Comparison of diversity between heavy and light fractions (SEATS station). (DOC)

S3 Table Comparison of diversity between heavy and light fraction (D001 station). (DOC)
