# Supplementary material for: Differential Incorporation of Carbon Substrates among Microbial Populations Identified by Field-Based, DNA Stable-Isotope Probing in South China Sea
Source: PLoS One. 2016 Jun 9;11(6):e0157178. doi: 10.1371/journal.pone.0157178 (PMC4900639; doi:10.1371/journal.pone.0157178)
Supplement: S1 Table — (DOC) [file pone.0157178.s001.doc]

**Differential incorporation of carbon substrates among microbial populations identified by field-based, DNA stable-isotope probing in South China Sea**

Yao Zhang^¶^*, Wenchao Deng^¶^, Xiabing Xie, Nianzhi Jiao*

State Key Laboratory of Marine Environmental Science & Institute of Marine Microbes and Ecospheres, Xiamen University, Xiamen 361101, China

*Corresponding author

E-mail: [yaozhang@xmu.edu.cn](mailto:yaozhang@xmu.edu.cn) (YZ); [jiao@xmu.edu.cn](mailto:jiao@xmu.edu.cn) (NJ)

^¶^These authors contributed equally to this work.

**S1 Table. Density fractions selected for 454-pyrosequencing analysis among the 12 density gradient fractions of each sample.**

| Station and depth | ^13^C-DOC | Heavy 1 (H1) | Heavy 2 (H2) | Middle (M) | Light (L) |
| --- | --- | --- | --- | --- | --- |
| SEATS 5 m | D-Glc | 2 | 4 | 8 | 11 |
|  | D-GlcN | 2 | 5 | 8 | 10 |
| SEATS 200 m | D-Glc | 2 | 4 | 7 | 10 |
|  | D-GlcN | 2 | 5 | 8 | 10 |
| D001 0 m | D-Glc | 2 | 5 | 7 | 11 |
|  | D-GlcN | 2 | 4 | 7 | 10 |
| D001 25 m | D-Glc | 2 | 5 | 8 | 10 |
|  | D-GlcN | 2 | 4 | 8 | 10 |
| D001 70 m | D-Glc | 2 | 5 | 7 | 10 |
|  | D-GlcN | 2 | 5 | 8 | 10 |
