# Supplementary material for: Differential Incorporation of Carbon Substrates among Microbial Populations Identified by Field-Based, DNA Stable-Isotope Probing in South China Sea
Source: PLoS One. 2016 Jun 9;11(6):e0157178. doi: 10.1371/journal.pone.0157178 (PMC4900639; doi:10.1371/journal.pone.0157178)
Supplement: S2 Table — (DOC) [file pone.0157178.s002.doc]

**Differential incorporation of carbon substrates among microbial populations identified by field-based, DNA stable-isotope probing in South China Sea**

Yao Zhang^¶^*, Wenchao Deng^¶^, Xiabing Xie, Nianzhi Jiao*

State Key Laboratory of Marine Environmental Science & Institute of Marine Microbes and Ecospheres, Xiamen University, Xiamen 361101, China

*Corresponding author

E-mail: [yaozhang@xmu.edu.cn](mailto:yaozhang@xmu.edu.cn) (YZ); [jiao@xmu.edu.cn](mailto:jiao@xmu.edu.cn) (NJ)

^¶^These authors contributed equally to this work.

**S2 Table. Comparison of diversity between heavy and light fractions (SEATS station)**

| Depth | Treatment | Shannon | | Simpson | | ACE | | Chao | |
| --- | --- | --- | --- | --- | --- | --- | --- | --- | --- |
|  |  | H2 | L | H2 | L | H2 | L | H2 | L |
| 5m | D-Glc | 4.904 | 5.454 | 0.072 | 0.018 | 3539 | 2527 | 2272 | 1692 |
|  | D-GlcN | 4.997 | 6.143 | 0.026 | 0.010 | 1919 | 5369 | 1312 | 3057 |
| 200m | D-Glc | 4.403 | 5.198 | 0.145 | 0.029 | 2404 | 3891 | 1650 | 2222 |
|  | D-GlcN | 5.279 | 5.937 | 0.015 | 0.013 | 2096 | 4424 | 1440 | 2629 |
