# Supplementary material for: Differential Incorporation of Carbon Substrates among Microbial Populations Identified by Field-Based, DNA Stable-Isotope Probing in South China Sea
Source: PLoS One. 2016 Jun 9;11(6):e0157178. doi: 10.1371/journal.pone.0157178 (PMC4900639; doi:10.1371/journal.pone.0157178)
Supplement: S3 Table — (DOC) [file pone.0157178.s003.doc]

**Differential incorporation of carbon substrates among microbial populations identified by field-based, DNA stable-isotope probing in South China Sea**

Yao Zhang^¶^*, Wenchao Deng^¶^, Xiabing Xie, Nianzhi Jiao*

State Key Laboratory of Marine Environmental Science & Institute of Marine Microbes and Ecospheres, Xiamen University, Xiamen 361101, China

*Corresponding author

E-mail: [yaozhang@xmu.edu.cn](mailto:yaozhang@xmu.edu.cn) (YZ); [jiao@xmu.edu.cn](mailto:jiao@xmu.edu.cn) (NJ)

^¶^These authors contributed equally to this work.

**S3 Table. Comparison of diversity between heavy and light fractions (D001 station)**

| Depth | Treatment | Shannon | | Simpson | | ACE | | Chao | |
| --- | --- | --- | --- | --- | --- | --- | --- | --- | --- |
|  |  | H2 | L | H2 | L | H2 | L | H2 | L |
| 0m | D-Glc | 5.342 | 5.058 | 0.014 | 0.017 | 2765 | 1744 | 1429 | 1002 |
|  | D-GlcN | 3.868 | 5.641 | 0.089 | 0.008 | 847 | 2740 | 501 | 1420 |
| 25m | D-Glc | 5.199 | 5.603 | 0.016 | 0.010 | 2630 | 2374 | 1249 | 1417 |
|  | D-GlcN | 3.508 | 5.901 | 0.118 | 0.006 | 649 | 2925 | 381 | 1668 |
| 70m | D-Glc | 5.610 | 5.941 | 0.011 | 0.005 | 2904 | 2703 | 1456 | 1738 |
|  | D-GlcN | 5.785 | 5.916 | 0.007 | 0.006 | 4385 | 4022 | 2201 | 1931 |
